# Clinical, molecular and cytopathological characterization of a Newcastle disease virus from an outbreak in Baghdad, Iraq

**DOI:** 10.1002/vms3.262

**Published:** 2020-03-31

**Authors:** Ahmed M. Al‐Shammari, Mohammed A. Hamad, Murtadha A. AL‐Mudhafar, Khansaa Raad, Aeser Ahmed

**Affiliations:** ^1^ Experimental Therapy Department Iraqi Center for Cancer and Medical Genetic Research Mustansiriyah University Baghdad Iraq; ^2^ Biotechnology and Environmental Center University of Fallujah Al‐Anbar Iraq; ^3^ Department of Microbiology Faculty of Veterinary Medicine University of Kufa Najaf Iraq

**Keywords:** lentogenic motif, pathogenicity, phylogenetic analysis, velogenic isolate

## Abstract

**Background:**

The frequent outbreaks of Newcastle disease virus (NDV) in Iraq pose a constant threat to commercial poultry, despite the introduction of routine vaccination programmes. Several factors, particularly stress factors and coinfections, might play a role in increasing NDV outbreaks in poultry species.

**Objectives:**

The current study was aimed to characterize an NDV isolate from an outbreak in North Baghdad, Iraq.

**Methods:**

Clinical pathogenicity of the isolate was determined experimentally in chickens. In vitro studies included cytopathological examination, as well as molecular and phylogenetic analyses.

**Results:**

Based on the clinical studies and pathogenicity indices (mean death time and intracerebral and intravenous pathogenicity indices), the isolate was characterized as velogenic (highly virulent). Reverse transcriptase polymerase chain reaction targeting the partial fusion protein gene of the NDV genome confirmed the detection. Partial sequencing of the hypervariable region of the fusion gene identified the presence of an avirulent (lentogenic) fusion protein motif (GRQGRL). Phylogenetic analysis of the new isolate along with previously known regional isolates revealed that the new isolate was related to genotype II strains. Additionally, sequence analysis indicated a distinct genetic lineage of the new isolate, which was related to some of the lineages identified in previous outbreaks in the Middle East.

**Conclusion:**

The current study offers essential information on the epidemiology, characteristics and diagnosis of NDV for disease control in Iraq. The isolate was found to belong to genotype II and possess an avirulent fusion protein motif.

## Graphical abstract

This study isolated NDV from an outbreak in North Baghdad, Iraq. The isolate was noted as velogenic by clinical experiment. Reverse transcriptase‐polymerase chain reaction (RT‐PCR) targeting the partial fusion‐protein gene of NDV genome, confirmed the virus. Partial sequencing of the fusion gene's hypervariable region reported a avirulent lentogenic fusion protein motif (GRQGRL) and phylogenetic analysis of the isolate with previously known 18 regional isolates, revealed it to be related to genotype II.

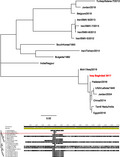

## 1 INTRODUCTION

Newcastle disease (ND) is extremely contagious, with devastating results for the poultry industry. Infection occurs through virus inhalation or ingestion and spreads from one bird to another, based on the availability of the virus in its virulent and infectious form (Alexander, 2001). Highly pathogenic avian influenza virus and Newcastle disease virus (NDV) have a significant impact on the poultry industry as they cause substantial economic losses and lead to high mortality in affected birds. These two diseases have been included in the Office International des Epizooties (OIE) list of diseases because of their seriousness (OIE, 2008). The causative agent of ND is a single‐stranded, negative‐sense RNA virus belonging to the genus *Avulavirus* of the family *Paramyxoviridae* (Mayo, 2002). The newly genetic characterization classifies NDV strains into genotypes I to XXI and several subgenotypes within a subset of genotypes (Dimitrov et al., 2019). Serologically, NDV strains comprise a single serogroup, the avian paramyxovirus serotype I (Russell & Alexander, 1983). Several studies have reported variations in the virulence of NDV (Alexander & Allan, 1973; Iorio, Borgman, Glickman, & Bratt, 1986). Based on the virulence, NDV can be categorized into four pathological forms namely, Doyle's form (viscerotropic velogenic), Beach's form (neurotropic velogenic), Baudette's form (mesogenic) and Hitchner's form (lentogenic) (Alexander & Allan, 1974). Biologically, these pathological forms are based on the virulence and nature of invading strains, as estimated by the intracerebral pathogenicity index (ICPI) and intravenous pathogenicity index (IVPI) (Piacenti, King, Seal, Zhang, & Brown, 2006).

Several ND outbreaks have been reported, despite the presence of intensive vaccination programmes, and the failures of the vaccination programmes were attributed to technical errors in the vaccination process due to the inexperience of the people involved (Spradbrow, 1991). However, in the last few years, antigenic variants that escape the protective immune responses engendered by the vaccines have been recognized as being responsible for such failures (Yang, Shieh, Lin, & Chang, 1999). Moreover, environmental factors like secondary immunocompromising infections may be reducing vaccine efficacy (Bolha et al., 2013).

In Iraq, numerous viral diseases still need to be controlled (Hamad, Al‐Shammari, Odisho, & Yaseen, 2016, 2018; Khudhair, Hasso, Yaseen, & Al‐Shammari, 2016). With respect to NDV, the Abu Ghraib 68 (AG68) vaccine strain displayed satisfactory and higher protection than those of other lentogenic strains, such as LaSota and B1, probably due to the close antigenic relationship between the local vaccine strain and the circulating NDV infection strains in Iraq (Jalob, Taha, Jumaa, Najim, & Salman, 2011). During the early 1990s, the AG68 strain disappeared for unknown reasons, and vaccine failure occurred from time to time among the flocks vaccinated with imported vaccines, resulting in substantial economic losses to the poultry industry (Jalob et al., 2011).

In Iraq, there is very little information regarding the differences between the invading NDV strains and the traditionally used vaccine strains. Recently, NDV strains have been isolated and diagnosed in several studies in Iraq (Al‐Shammari, Al‐Nassrawei, & Kadhim, 2014; Al‐Shammari, Al‐Nassrawei, & Murtadha, 2014). The present study was designed to characterize the causative agent of the recent outbreaks around Baghdad, Iraq. The study was carried out using clinical and cytopathological examinations and phylogenetic analyses to evaluate differences between the invading strains and the traditionally used vaccine strains. Information regarding the molecular basis of pathogenicity may help in the identification of new invading strains and in studying disease epidemiology through the detection of slight antigenic variations such as differences at a single amino acid level.

## 2 MATERIALS AND METHODS

### 2.1 Clinical samples

The present study investigated an outbreak of suspected NDV among vaccinated chickens at a broiler breeder farm during the year 2017 in North Baghdad, Iraq. The results of post‐mortem inspection were presented to the Department of Veterinary Pathology, University of Al‐Anbar College of Veterinary Medicine, Al‐Anbar, Iraq, during the year, 2017. The chickens were diagnosed through a gross necropsy examination. Tissue samples from the trachea and other internal organs (spleen, liver, heart and brain) were collected and processed for virus isolation. High mortality rates (>80%) were detected in sick birds, which showed signs of a respiratory infection, agitation and digestive distress.

### 2.2 NDV isolation

Tissue homogenates were prepared in phosphate‐buffered saline (PBS) containing penicillin (2,000 U/mL) and streptomycin (500 µg/ml) and centrifuged at 2,012 *g* for 15 min. The supernatant (0.1 ml) was inoculated into the allantoic cavity of five embryonated (10‐day old) chicken eggs (OIE, 2008). The eggs were incubated at 37°C for 4–7 days with daily observations. The allantoic fluid was harvested and tested using a haemagglutination (HA) test to detect the haemagglutinating virus. Samples that tested positive in the HA test were further examined for the presence of NDV using a specific anti‐NDV monoclonal antibody (Santa Cruz Biotechnology, Inc.). Aliquots of the NDV‐positive allantoic fluid were stored at −86°C for conventional polymerase chain reaction (PCR) and sequencing analysis.

### 2.3 Mean death time (MDT) test

To perform this test, NDV isolates were serial diluted 1:10 with PBS. For each dilution, five embryonated eggs (10‐day old) were inoculated into the allantoic cavity with a diluted virus (0.2 ml) or PBS, which served as a control. After 24 hr of incubation, the eggs were checked every 2 hr for embryonic mortality for an additional 96 hr.

### 2.4 ICPI test

The ICPI test was performed to measure the virulence of the virus, by intracerebrally injecting 0.05 ml of 1:10 diluted virus (containing allantoic fluid) into 1‐day‐old specific pathogen‐free chickens, which were examined for 8 days following the injection. In this period, healthy chickens were scored as 0, whereas sick and dead chickens were scored as 1 and 2, respectively. The ICPI values were calculated based on the average ICPI score of 10 chickens (OIE, 2008).

### 2.5 IVPI test

IVPI was estimated in 6‐week‐old chickens. The chickens were inoculated intravenously with 0.1 ml of 10‐fold diluted virus‐containing allantoic fluid and were examined for 10 days. Healthy chickens were scored as 0, whereas sick, paralyzed and dead chickens were scored as 1, 2 and 3, respectively. The IVPI value was calculated based on the average IVPI score of 10 birds.

### 2.6 Experimental design

In all, 60 unvaccinated 1‐day‐old broiler chicks (Ross 308), obtained from a commercial supplier, were segregated into two equal groups. One group was vaccinated with the LaSota vaccine (Intervet, Inc.) when the chickens were 12‐day old and re‐vaccinated at 22 days via drinking water, whereas the second group was not vaccinated. Birds in each group were intranasally challenged on day 32 with a local virulent virus (at a titre of 10^6.5^ EID_50_) isolated during the outbreak. Clinical signs and mortality were documented daily.

### 2.7 Cytopathic effects of NDV isolates in cell culture

The NDV‐infected and non‐infected (control) Vero and HeLa cell lines were supplied by the Cell Bank Unite, Experimental Therapy Department, Iraqi Center for Cancer and Medical Genetic Research, Mustansiriyah University. Cells were imaged at regular intervals every 24 hr for 72 hr and then fixed in 4% paraformaldehyde prepared in phosphate buffer (pH 7.4) for 10 min at 4°C. The cells were examined using an inverted microscope (Leica Microsystems GmbH). Apoptotic cells were identified based on their morphological characteristics, including plasma membrane blebbing, cytoplasmic vacuolation and chromatin condensation.

### 2.8 Viral RNA preparation

The NDV RNA was extracted directly from the infected allantoic fluid using the Magnesia^®^ Cultured Cell and Tissue Total RNA Extraction Kit (Anatolia Geneworks), with the Magnesia^®^ 16 magnetic bead extraction system (Anatolia Geneworks) according to the manufacturer's instructions.

### 2.9 Reverse Transcriptase‐polymerase chain reaction (RT‐PCR)

We followed a previously described RT‐PCR protocol (Cattoli & Monne, 2009) for genotyping and pathotyping of APMV‐1 isolates. A primer pair targeting the hypervariable region of the fusion gene (forward NDV: 5′‐TACACCTCATCCCAGACAGG‐3′; reverse NDV: 5′‐AGTCGGAGGATGTTGGCAGC‐3′) was used to amplify a 300‐base pair (bp) fragment. For RT‐PCR, RNA (5 µl) was denatured and used as a template. RT‐PCR was performed according to the manufacturer's instructions (Agilent Technologies Stratagene). Briefly, RT‐PCR reactions were run on an Agilent 8800 gradient PCR. The reaction mixture (50 µl) contained RNase‐free water (17.5 µl), Herculase II RT‐PCR 2× Master Mix (25 µl), primers (1 µl each) and AffinityScript RT/RNase Block (0.5 µl). Reverse transcription was carried out at 45°C for 5 min with an initial denaturation step at 72°C (1 min). RT‐PCR comprised 40 cycles of denaturation (92°C, 20 s), annealing at temperatures (49.5°C, 49°C and 48.5°C for 20 s sequentially), extension (72°C, 30 s) and a final extension (72°C, 3 min). The reaction mixture (50 µl) was loaded with a loading dye (5 µl) into the wells of an agarose gel (1%, w/v) containing ethidium bromide and electrophoresed, followed by visualization using a VISION gel documentation system (Scie‐Plas).

### 2.10 Sequencing

Sequencing of purified PCR products was performed in both directions at the National Instrumentation Center for Environmental Management, College of Agriculture and Life Sciences, Seoul National University, using a 3730XL DNA analyzer (Applied Biosystems, Inc.). The complementary sequences were aligned using the ApE software v2.0.55 (http://jorgensen.biology.utah.edu/wayned/ape). The nucleotide sequence of the *F* gene of the NDV isolate was submitted to GenBank under accession number MH638994.1.

### 2.11 Phylogenetic analysis

Phylogenetic analysis was performed to determine the evolutionary relationships between the new local isolate and 18 reference NDV strains that chose according to the geographical location and the relation in the NCBI blast result. A phylogenetic tree was constructed by the neighbour‐joining method using Geneious R10 v10.2.6 (https://www.geneious.com). Accession numbers of the sequences of the NDV strains are presented in Table 1.

**Table 1:** Reference sequences of NDV strains—with diverse genotypes—included in the study for comparative phylogenetic analysis

No.	NDV strain	Accession number	Genotypes
1	India Nagpur	KX372711_1	XIIIb
2	Moh1/Iraq/2016	MH407202_1	II
3	Iraq Abu Ghraib/Baghdad 2017	MH638994.1	II
4	Pakistan/2016	MG686585_1	II
5	Tamil Nadu/India	HM357251_1	II
6	Egypt/2016	MH559344_1	II
7	China/2014	KY788672_1	II
8	USA/LaSota/1946	MH392212_1	II
9	Jordan/2004	KY212127_1	II
10	South Korea/1993	KY042143_1	XX
11	Iran/Tehran/2014	MF179101_1	Unknown
12	Bulgaria/1982	KY042126_1	VI
13	Belgium/2018	MH432252_2	VIIi
14	Turkey/Adana‐7/2012	KP271979_1	VIIi
15	Jordan/2018	MH614933_1	VII.2
16	Iran/SMV‐5/2012	KU201412_1	VIIl
17	Iran/SMV‐8/2013	KU201415_1	VIIl
18	Iran/SMV‐7/2013	KU201414_1	VIIl
19	Iran/SMV‐6/2012	KU201413_1	VIIl

## 3 RESULTS

### 3.1 Viral pathogenicity

Based on the pathogenicity indices, the NDV isolate (designated Abu Ghraib/2017) was characterized as virulent (velogenic). The studied NDV isolate showed an MDT score of less than 50 hr, whereas the ICPI and IVPI values were 1.96 and 2.56, respectively. The unvaccinated broiler chicks infected with the Abu Ghraib/2017 isolate developed clinical signs of the disease, such as severe depression within 72‐hr post‐inoculation, and had a high mortality rate (97%) during the course of the experiment. Meanwhile, the vaccinated birds demonstrated no signs of clinical disease and showed a low mortality rate (3%). The main necropsy findings were air sac culitis, tracheitis, haemorrhages in the proventriculus, and also the presence of visceral intestinal and caecal lesions.

### 3.2 Cytopathic effects of the NDV isolate

**Figure 1:**
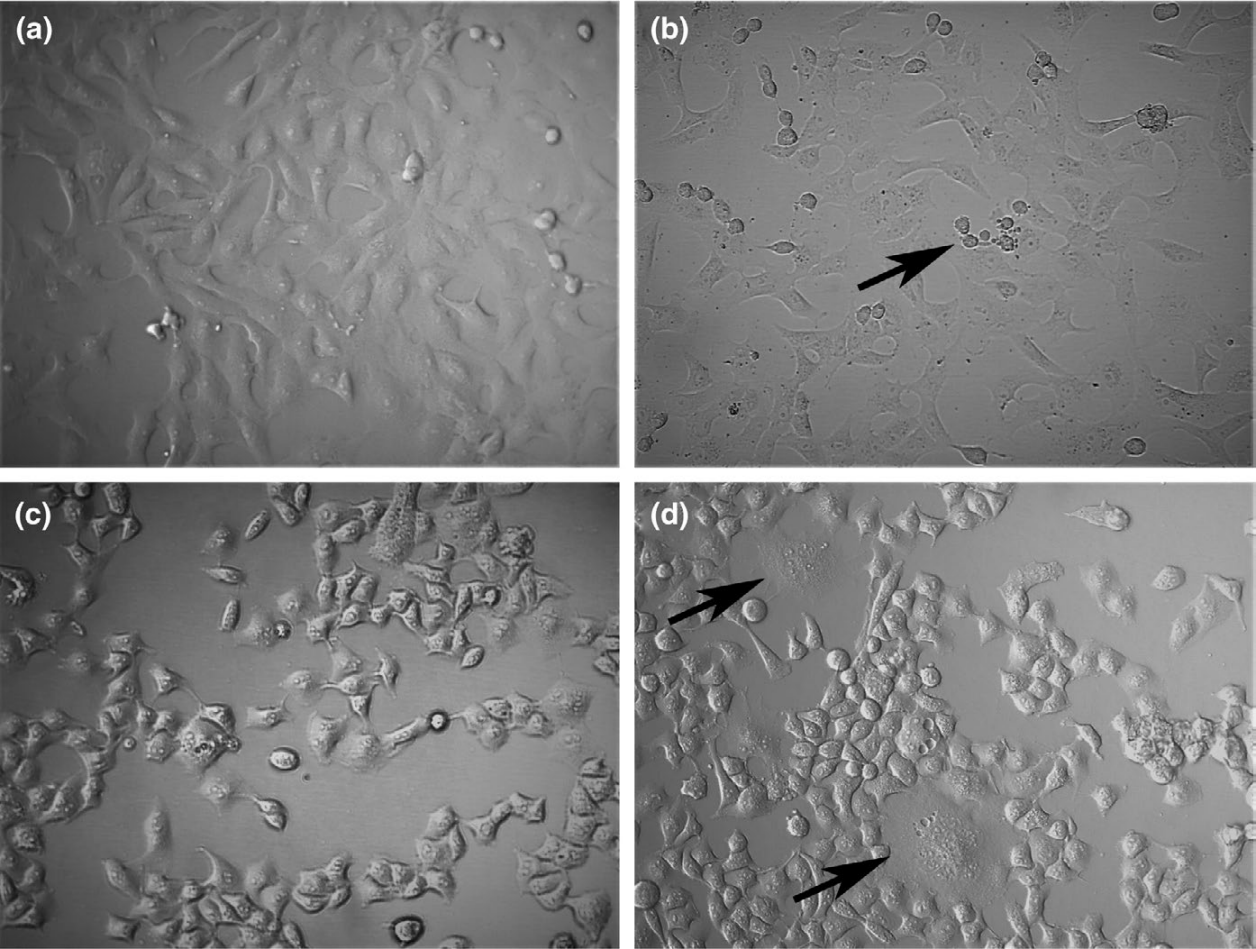
Cytopathic effect of Newcastle disease virus (NDV) Abu Ghraib/2017 after 24 hr of infection. (a) Non‐infected Vero cells. (b) Infected Vero cells displaying a decrease in viability and the presence of rounded cells (black arrow). (c) Non‐infected HeLa cells. (d) Infected HeLa cells displaying significant syncytia formation (black arrow), along with cell membrane blebbing and cytoplasm vacuolation. Magnification: 20×

The NDV‐infected and non‐infected Vero and HeLa cell lines were examined to evaluate the cytopathic effects of NDV Abu Ghraib/2017. After 24 hr of infection, a decrease in the viability was noted in the NDV‐infected Vero cells; rounded cells—the main sign of infection—were observed (Figure 1a and b). Compared with that of the control cells, the infected HeLa cells exhibited a slight decrease in viability and significant syncytia formation—which is the characteristic of NDV infection in cell culture—along with membrane blebbing and cytoplasm vacuolation (Figure 1c and d).

### 3.3 Molecular detection of NDV

**Figure 2:**
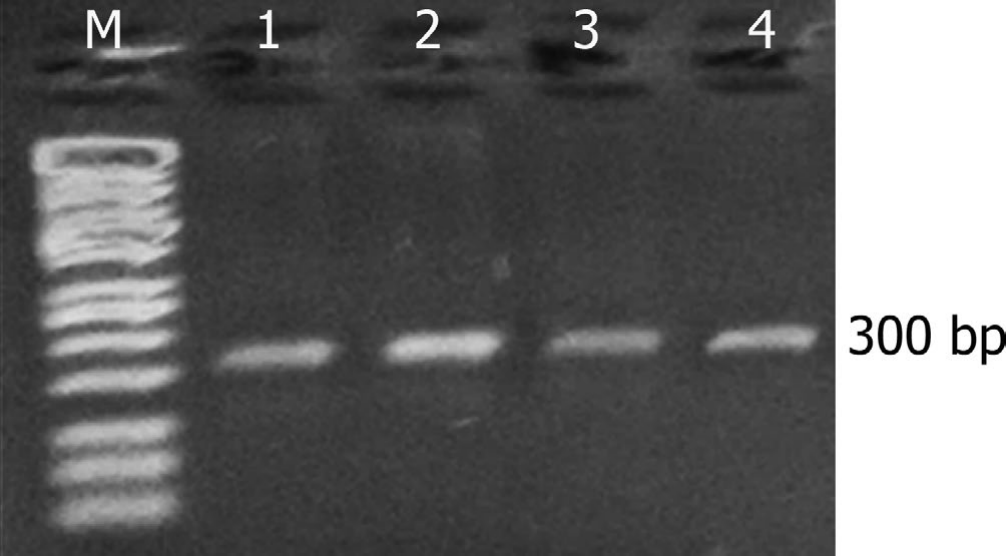
Electrophoretic pattern of *F* gene PCR products, exhibiting a 300‐bp band corresponding to that of the *F0* gene of NDV. M: 100–10,000‐bp marker; lanes 1–4: Abu Ghraib/2017 isolates

The results of the RT‐PCR confirmed the results of the laboratory tests, indicating that all the examined field samples were positive for NDV. The results of RT‐PCR using a primer set against the partial *F* gene revealed bands of the expected size (Figure 2). The NDV Abu Ghraib/2017 isolate exhibited a specific 300‐bp band.

### 3.4 Sequencing

Analysis of the predicted amino acid sequence, which was based on the partial sequence of the hypervariable region of the gene encoding the fusion (F0) protein in the Abu Ghraib/2017 isolate, showed the presence of an avirulent (lentogenic) motif (GRQGRL) at the F0 protein cleavage site.

### 3.5 Phylogenetic analysis

**Figure 3:**
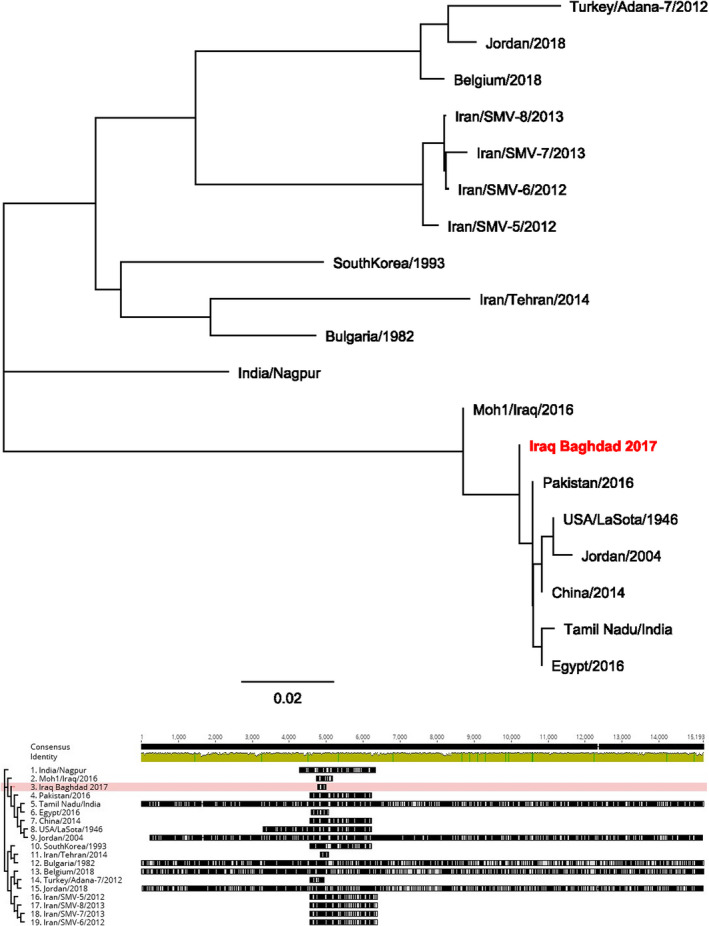
Phylogenetic analysis of the NDV/chicken/Iraq/Baghdad/Abu Ghraib/2017 isolate, based on the nucleotide sequence of the 300‐bp region of the *F0* gene. (a) Neighbour‐joining phylogenetic tree of the nucleotide sequences of the partial fusion gene fragment from the Abu Ghraib/2017 isolate and reference strains from GenBank. (b) Partial nucleotide sequences of the *F* gene from the NDV isolates

A phylogenetic tree was constructed using the partial sequence of the *F* gene (accession number: MH638994.1) of the Abu Ghraib/2017 isolate and 18 different NDV reference sequences to determine the origin of Abu Graib/2017 (Figure 3). The results of the phylogenetic analysis revealed that the Abu Ghraib/2017 isolate was very closely related to previously reported NDV isolates from Iraq, especially Moh1/Iraq/2016 and MH407202. Moreover, Abu Ghraib/2017 was grouped with strains belonging to genotype II from Iraq (Moh1/Iraq/2016 and MH407202) (Hussein & Khammas, 2019), Jordan (Jordan/2004 and KY212127) (Chrzastek et al., 2017) and Egypt (Egypt/2016 and MH559344) (Kilany, Ali, Bazid, Zain El‐Abideen, & ElSayed, 2015) as well as LaSota vaccine strain (Figure 3a and b).

## 4 DISCUSSION

In Iraq, frequently occurring NDV outbreaks pose a constant threat to commercial poultry, despite routine vaccination. The suggested reason for the NDV outbreaks in vaccinated populations could be due to environmental factors like secondary immunocompromising infections reducing vaccine efficacy (Bolha et al., 2013). Moreover, this poor flock immunity along with inadequate vaccination practices could lead to ineffective vaccination and reoccurring of NDV outbreaks (Yang et al., 1999). Several studies have been conducted to control the disease through the development of antiviral agents, such as the leaf extract of a natural herb, *Olea europaea* (Salih, Odisho, Al‐Shammari, & Ibrahim, 2017) and the phytochemicals arctigenin and limonin (Abd Ali, Majeed, & Hamoody, 2019; Jassim, Al‐Shammari, & Abd Al‐Hameed, 2019).

The pathogenicity indices reveal that Abu Ghraib/2017 was a virulent isolate, as it showed an MDT score of less than 50 hr. The ICPI and IVPI values observed were 1.96 and 2.56, respectively, which are the characteristic features associated with velogenic NDV strains as per the OIE (OIE, 2008).

Surprisingly, the molecular analysis of the Abu Ghraib/2017 isolate—which was clinically and experimentally proven to be virulent—revealed the presence of an avirulent (lentogenic) fusion protein motif (GRQGRL) that corresponded with the sequence of the hypervariable region of the fusion gene. Similarly, molecular characterization of the partial fusion gene of the Malaysian virulent isolates from clinical cases revealed that a velogenic isolate (accession number: MB016/07) also possessed the common avirulent GRQGRL motif (Berhanu, Ideris, Omar, & Bejo, 2010). Moreover, our findings were similar to those reported in China (Qin et al., 2008), as they found that most genotype II isolates holding the lentogenic motif 112G‐R‐Q‐G‐R‐L117 were virulent. They described the presence of a high degree of homology between genotype II isolates and the reference strain LaSota which exactly matches our findings. They assumed that the F gene cleavage site motifs analysis were not enough to differentiate between low‐virulence and virulent NDV strains and that the virulence determination may rely on the role of other genes of the viral genome. Hence, NDV characterization requires both the biological tests and the analyses of the F gene.

The F protein precursor is a protein of 553 amino acids which has to be activated by cleavage into F1 and F2 subunits by host cell proteases. The amino acid sequence at the proteolytic cleavage site determines substrate specificity and influences viral virulence. Alteration of a monobasic amino acid sequence at the F protein cleavage site to a polybasic motif increased virulence of a lentogenic NDV to a mesogenic phenotype (Heiden et al., 2014; Paldurai et al., 2014).

The phylogenetic analysis showed that the Abu Ghraib/2017 isolate was identical to another Iraqi isolate, isolated from Karbala, a place that is 180 km away from the location of isolation of Abu Ghraib/2017 isolation. Furthermore, the phylogenetic tree displayed similarities with regional velogenic NDV strains from Jordan and Egypt (Chrzastek et al., 2017; Kilany et al., 2015). The phylogenetic tree constructed in the current study revealed that the newly characterized NDV strain belonged to the genetic group designated as genotype II. The analysis also demonstrated that the Abu Ghraib/2017 isolate was grouped with the LaSota strain in the same genotype II cluster.

The close genetic distance between the recent virulent NDV Abu Ghraib/2017 isolate and the LaSota vaccine strain suggests that the recent virulent could represent a revertant from the vaccine. Furthermore, there are documented reports that class II NDV isolates of low virulence have been shown to be able to evolve naturally to a virulent phenotype (Alexander, 2011), as just a few point mutations can cause the emergence of a virulent form of NDV (Collins, Franklin, Strong, Meulemans, & Alexander, 1998; Dimitrov et al., 2016). Also, this might explain why vaccination with LaSota gave good protection results in the current experiment due to high antigenic similarity which induces more specific neutralizing antibodies.

The isolate, Abu Ghraib/2017, was tested for cytopathic effects in cell culture studies, which displayed significant cytological deterioration such as cell rounding and detachment, as well as syncytia formation, which is a characteristic sign of NDV infection in cell culture. As a paramyxovirus, NDV exerts cytopathic effects in different cell lines (Ahmed M Al‐Shammari, Yaseen, & Alwan, 2012; Csatary, 1971). Moreover, cytolysis, secondary to viral replication, is thought to induce the cytopathic effects (Fiola et al., 2006). Inhibition of glycolysis has been suggested as a mechanism for NDV‐induced cytopathic effects (Al‐Shammari, Abdullah, Allami, & Yaseen, 2019). In the present study, morphological changes during apoptosis, such as cell membrane blebbing and cytoplasm vacuolation, were detected after 24 hr of NDV infection. Several studies have reported DNA fragmentation and caspase‐3 upregulation in NDV‐infected cells (Al‐Shammari, Humadi, Al‐Taee, Al‐Atabi, & Yaseen, 2015; Al‐Shammari et al., 2012; Al‐Shammary, Hassani, & Ibrahim, 2014).

In conclusion, a virulent (velogenic) NDV strain, named Abu Ghraib/2017, was isolated during an ND outbreak in North Baghdad, Iraq, and characterized in this study. The isolate was found to belong to genotype II and possess an avirulent fusion protein motif. Microscopic examination revealed that the isolate induced a marked cytopathic effect in susceptible cells. Virulent NDV strains, which are still regularly isolated in Iraq, cause economic losses in the poultry industry; therefore, controlling this disease is extremely important.

## CONFLICT OF INTEREST

The authors declare no conflicts of interest.

## AUTHOR CONTRIBUTIONS

**Ahmed Majeed Al‐Shammari:** Conceptualization; Data curation; Formal analysis; Investigation; Methodology; Project administration; Software; Supervision; Validation; Visualization; Writing‐original draft; Writing‐review & editing. **Mohammed A Hamad:** Conceptualization; Data curation; Formal analysis; Investigation; Methodology; Writing‐original draft. **Murtadha A AL‐Mudhafar:** Investigation; Methodology; Project administration; Resources; Validation; Visualization. **Khansaa Raed:** Data curation; Investigation; Methodology. **Aesar Ahmed:** Data curation; Investigation; Methodology.

## ETHICAL STATEMENT

The authors confirm that the ethical policies of the journal, as noted in the journal's author guidelines, have been adhered to, and approval has been obtained from the appropriate ethical review committee. The guidelines proposed by the US National Research Council regarding the Care and Use of Laboratory Animals were followed.
